# Exploring the genetic landscape of nitrogen uptake in durum wheat: genome-wide characterization and expression profiling of NPF and NRT2 gene families

**DOI:** 10.3389/fpls.2023.1302337

**Published:** 2023-11-09

**Authors:** Guglielmo Puccio, Rosolino Ingraffia, Dario Giambalvo, Alfonso S. Frenda, Alex Harkess, Francesco Sunseri, Francesco Mercati

**Affiliations:** ^1^ Department of Agricultural, Food and Forestry Sciences, University of Palermo, Palermo, Italy; ^2^ Institute of Biosciences and BioResources (IBBR), National Research Council, Palermo, Italy; ^3^ HudsonAlpha Institute for Biotechnology, Huntsville, AL, United States; ^4^ Department Agraria , University Mediterranea of Reggio Calabria, Reggio Calabria, Italy

**Keywords:** durum wheat, nitrogen, N uptake, nitrate transporters, NPF and NRT2 family, Nitrogen Use Efficiency (NUE), N uptake, Weighted Gene Co-expression Network Analysis (WGCNA)

## Abstract

Nitrate uptake by plants primarily relies on two gene families: Nitrate transporter 1/peptide transporter (NPF) and Nitrate transporter 2 (NRT2). Here, we extensively characterized the NPF and NRT2 families in the durum wheat genome, revealing 211 NPF and 20 NRT2 genes. The two families share many Cis Regulatory Elements (CREs) and Transcription Factor binding sites, highlighting a partially overlapping regulatory system and suggesting a coordinated response for nitrate transport and utilization. Analyzing RNA-seq data from 9 tissues and 20 cultivars, we explored expression profiles and co-expression relationships of both gene families. We observed a strong correlation between nucleotide variation and gene expression within the NRT2 gene family, implicating a shared selection mechanism operating on both coding and regulatory regions. Furthermore, NPF genes showed highly tissue-specific expression profiles, while NRT2s were mainly divided in two co-expression modules, one expressed in roots (NAR2/NRT3 dependent) and the other induced in anthers and/ovaries during maturation. Our evidences confirmed that the majority of these genes were retained after small-scale duplication events, suggesting a neo- or sub-functionalization of many NPFs and NRT2s. Altogether, these findings indicate that the expansion of these gene families in durum wheat could provide valuable genetic variability useful to identify NUE-related and candidate genes for future breeding programs in the context of low-impact and sustainable agriculture.

## 1 Introduction

Nitrogen (N) is a crucial nutrient for plant growth and development. Suboptimal nitrogen utilization can lead to diminished yields and significant environmental repercussions. Excessive or misapplied nitrogen fertilizers often lead to an increased risk of nitrogen escaping into the environment through processes like denitrification, leaching, or volatilization. This contributes to higher levels of nitrate in both surface and groundwater, as well as the release of N_2_O and NH_3_ into the atmosphere. Therefore, improving the efficiency of nitrogen utilization is crucial to address issues such as environmental degradation, climate change, and food security (Javed et al., 2022). Despite valuable research efforts in this field and the development of various technologies (i.e., slow-release fertilizers, inhibitors for nitrification and urease, fertigation, and advanced precision agriculture techniques) nitrogen efficiency remains relatively low for many crops, particularly for cereals where it typically ranges between 25% and 50% of the applied nitrogen (Giambalvo et al., 2018; Javed et al., 2022). This can be attributed to the complexity of Nitrogen Use Efficiency (NUE), which involves a multitude of factors related to agronomy, physiology, and molecular biology. Nitrate (NO_3_^-^) is one of the major N-forms taken up by plants from the soil. NO_3_^-^ availability in the soil is highly variable and its uptake is governed by at least two transport systems, depending on soil NO_3_^-^ concentrations: the low affinity NO_3_^-^ transport (LATS) and the high affinity NO_3_^-^ transport (HATS) systems. LATS is mediated by the NO_3_^-^ transporter 1/peptide transporter (NRT1/NPF) family, which comprises a diverse array of membrane transport proteins found within multiple cell types and tissues, whereas HATS is facilitated by the NRT2 family, and is specific for NO_3_^-^. These two transport systems are responsible for the uptake of NO_3_^-^ at different range of concentrations from millimolar to micromolar. N uptake is an important component of NUE, defined as the total biomass (or yield) per unit of N supplied (Moll et al., 1982), it is a complex trait influenced by interacting environmental factors and controlled by gene networks involved in N uptake, assimilation, and remobilization. NUE is divided in two main components, the Nitrogen Uptake Efficiency (NUpE), referred to the ability of the plant to take up N from the soil, and the Nitrogen Utilization Efficiency (NUtE), which encompasses the ability of the plant to assimilate, transfer, and utilize N to the harvestable part of the crop (Good et al., 2004; Xu et al., 2012).

The NPF and NRT2 families differ in both their structure and copy number across angiosperms. The NPF family harbors a conserved structural arrangement consisting of twelve transmembrane domains (TM) connected by short peptides and a central hydrophilic loop of about 90 amino acids between the sixth and the seventh TM domains (Wang et al., 2018b). They were previously known as NRT1s (NO_3_^-^ transporters) and/or PTRs (peptide transporters) depending on their first discovered substrates. Based on a wide multi-species phylogenetic analysis, Léran et al. (2014) proposed a unified nomenclature for the NO_3_^-^ transporter/Peptide transporter family (NPF), defining eight subfamilies (NPF1-8). The first NPF gene member isolated in plants and one of the most studied is the *Arabidopsis thaliana* NPF6.3 (*AtNPF6.3*), previously known as *CHL1/AtNRT1.1*. It is considered a dual-affinity NO_3_^-^ transporter contributing to root NO_3_^-^ uptake at both low (LATS) and high (HATS) NO_3_^-^ availability, acting also as an NO_3_^-^ sensor or ‘transceptor’ (Liu et al., 1999; Gojon et al., 2011; Xuan et al., 2017). *AtNPF6.3* can also act as a chlorate transporter (per the old name *CHL1* was awarded) when NO_3_^-^ is less available and as an auxin transporter, a process negatively regulated by NO_3_^-^ (Mounier et al., 2014; Maghiaoui et al., 2020; Meier et al., 2020). The interaction between auxin and NO_3_^-^ is associated to NO_3_^-^ sensing and it is involved in the regulation of N-dependent root development (Bouguyon et al., 2015). NPF proteins can transport a high number of different substrates other than NO_3_^-^, including phytohormones such as ABA and auxin, but also peptides, potassium, and secondary metabolites (Chiba et al., 2015; Tal et al., 2016; Kanstrup and Nour-Eldin, 2022). Although the NPF family is often involved in the LATS, many members also show high affinity transport in many species such as *ZmNPF6.6* and *MtNPF6.8* in maize and *Medicago truncatula*, respectively (Bagchi et al., 2012; Wen et al., 2017).

Land plant genomes typically contain a higher number of NPF/PTR genes compared to bacteria, animals, and algae, with 20 members in the moss *Physcomitrella patens*, 52 members in *Arabidopsis thaliana* and even more members in polyploid species such as *Brassica napus* (199) and *Triticum aestivum* (331) (Bajgain et al., 2018; Longo et al., 2018). In *Brassica napus*, allopolyploidy greatly contributed to the gene family expansion of the NPF family (Wen et al., 2020). A recent characterization of the NPF and NRT2 families in bread wheat also showed an expansion of these families (331 and 46, respectively) mainly due to tandem and segmental duplication (Bajgain et al., 2018; Li et al., 2021). The retention of multiple gene copies, after duplication, can be associated with the acquisition of new beneficial functions or the reduction of their full capacity, compared to that of the single-copy ancestral gene (Lynch and Conery, 2000). The high number of NPF genes in allopolyploid species suggested that the transporters encoded by these genes may have evolved for new unknown roles in plants (Corratg’e-Faillie and Lacombe, 2017; Longo et al., 2018). Thus, exploration for novel functions within these large gene families in polyploid crops is necessary. The NRT2 genes are primarily involved in HATS, and mainly active in roots, although some members are expressed in other tissues such as seeds or leaves to allow NO_3_^-^ remobilization and storage (Chopin et al., 2007; Miller et al., 2007). Seven members were characterized in *Arabidopsis thaliana*, while five were detected in *Oryza sativa*. Similarly to the NPF family, a higher number of NRT2 members were discovered in allopolyploid species such as *Triticum aestivum* and *Brassica napus* with 47 and 17 genes, respectively (Tong et al., 2020; Li et al., 2021). This family was deeply studied in *Arabidopsis thaliana*, *AtNRT2.1* resulted the most studied member due to its main role in high affinity NO_3_^-^ uptake in roots (Li et al., 2007). The NRT2 genes are usually identified based on the sequence homology to known NO_3_^-^ transporters, then their functions are predicted through gene expression analysis and heterologous expression in *Xenopus* oocytes. Nonetheless, several studies on monocot species such as wheat and rice have highlighted high sequence divergence with dicot species, making it hard to directly infer gene functions relying only on sequence identity (Plett et al., 2010; Pellizzaro et al., 2015; Wang et al., 2019a). Therefore, the utilization of multi-tissue and -condition expression data become mandatory to fully characterize these genes in monocot crops, mainly in the allopolyploids.

The hexaploid bread wheat (*Triticum aestivum* L.; genome AABBDD) is among the most important global crop species, shaped heavily by polyploidy and hybridization between the tetraploid durum wheat (*Triticum turgidum* L.; genome AABB) and *Aegilops tauschii* (genome DD). The NPF and NRT2 gene families have been investigated mainly in bread wheat, exploring their expression levels under different abiotic stresses, such as drought, salt and N deficiency, in response to Arbuscular Mycorrhizal Fungi (AMF), and in several tissues and development stages (Buchner and Hawkesford, 2014; Duan et al., 2016; Tian et al., 2017; Bajgain et al., 2018). Recently, the increase of grain NO_3_^-^ uptake through the *TaNRT2.5* overexpression, localized in the grain cell tonoplast, was reported (Li et al., 2020). Many other studies highlighted improved crop yield, shoot biomass, and N uptake when NPF or NRT2 genes were overexpressed (Hu et al., 2015; Fan et al., 2016; Sol et al., 2019; Wang et al., 2021). Furthermore, the nucleotide variability in protein-coding regions of the NPF genes seems to affect NUE related traits such as yield and shoot N content (Li et al., 2021). These findings suggested that further efforts in the detection and functional characterization of both gene families may greatly aid the selection of N-use efficient wheat cultivars. The high wheat genetic variability, the high number of duplicated genes, and its economic relevance make this plant a key species for the screening of potentially beneficial genes.

Compared to bread wheat, much less is known about the phylogenetic diversity, evolution, and expression of the NPF and NRT2 gene families in tetraploid durum wheat, which is an important crop in the Mediterranean basin (Hawkesford, 2017; Hawkesford and Griffiths, 2019; Lupini et al., 2021). The detection of key genes involved in NO_3_^-^ transport is a primary goal for NUE improvement, and a gene family comparison between durum and bread wheat can elucidate the impact of polyploidy on NUE components. Hence, it is crucial to undertake a thorough characterization and annotation of nitrate transporters in the durum wheat genome. In this study, we have identified and analyzed both NPF and NRT2 gene families, exploring their phylogenetic relationships, gene and protein structures, regulatory elements, and expression profiles within the durum wheat genome.

## 2 Materials and methods

### 2.1 NPF and NRT2 identification in durum wheat genome

To identify NPF and NRT2 genes in the durum wheat genome, the protein sequences of NPF and NRT2 genes of *Arabidopsis thaliana*, barley (*Hordeum vulgare*), maize (*Zea mays*), rice (*Oryza sativa*), and bread wheat (*Triticum aestivum*) were downloaded from Ensembl plants (http://plants.ensembl.org/). These sequences were used for a BLASTP search against the entire durum wheat proteome, also downloaded from Ensembl plants, using an e-value threshold of 1e^-10^ and a minimum sequence identity of 50%. The durum wheat BLASTP best hits were then used as input for HMMER3 (Mistry et al., 2013), using the hmmscan command and the ‘Proton-dependent oligopeptide transporter family’ (IPR000109) HMM profile with an e-value cut-off of 1e^-^05 for the NPF genes. Furthermore, Pfam (Bateman et al., 2004) and NCBI protein sequence analysis tools were used to check that all the NPF protein sequences belonged to the PTR2 family (PF00854) and that all the NRT2 protein sequences contained the NCBI conserved domain PLN00028. The final set of genes was then used to identify homologous groups. These were defined through a reciprocal BLASTN using nucleotide sequence identity >95%.

### 2.2 Motif discovery, TF binding site, CREs prediction, and gene structure analysis

Gene structure of both TdNPF and TdNRT2 family members using Webscipio2 was analyzed (Hatje et al., 2011). The Multiple Em for Motif Elicitation (MEME) suite (Bailey et al., 2015) was used to identify conserved motifs with the following parameters: classic mode algorithm, 6 and 100 for minimum and maximum motif width, and a maximum number of 30 motifs per sequence. Conserved motifs were further analyzed through the NCBI protein domain search tool (https://www.ncbi.nlm.nih.gov/Structure/cdd/wrpsb.cgi) and the Conserved Domain Database (CDD), using an e-value threshold of 0.01. Transmembrane helices and protein localization prediction was performed using the TMHMM2.0 tool (Krogh et al., 2001) and both WoLF PSORT (Horton et al., 2007) and PProwler1.2 (Hawkins and Bodén, 2006), respectively. Chromosome location was extracted from the durum wheat genome annotation v1.0 and then displayed using the MG2C online tool (Chao et al., 2021). Significantly enriched chromosomal locations for both NPF and NRT2 were detected with ShinyGO (Ge et al., 2020) using a sliding window size of 6Mb and an FDR cutoff of 1e-05. The same tool was used to perform a GO enrichment analysis of both TdNPF and TdNRT2 genes.

Transcription Factor (TF) binding site prediction was performed on the promoter region using the binding site prediction tool of the Plant Transcription Factor Database (http://plantregmap.gao-lab.org/binding_site_prediction.php) with a p-value threshold of 1e^-^06 and the *Triticum aestivum* orthologs. The UniProtKB database (www.uniprot.org) was then used to extract protein domain information and annotation of the predicted TFs. Cis-regulatory Elements (CREs) in upstream promoter regions (− 2000 bp) of *TdNPFs* and *TdNRT2s* were predicted using PlantCARE (Lescot et al., 2002).

### 2.3 Collinearity and gene duplications analysis

The intraspecific collinearity was analyzed using both TdNPF and TdNRT2 gene sets. A reciprocal BLASTP was performed using an e-value threshold of 1e^-10^. MCScanX was used to evaluate collinearity and duplication events using an e-value threshold of 1e-^05^ and a match score of 50. MCScanX was also used to display the collinear blocks among five *Poaceae* species selected on the knowledge about the genesis of both durum and bread wheat (Kimber and Feldman, 1987; Matsuoka, 2011) (*Aegilops speltoides* Tausch: closer to B genome, *Triticum urartu*: A genome, *Triticum durum*: A and B genomes, *Triticum aestivum*: A, B and D genomes, and *Aegilops tauschii*: D genome). Collinear blocks between species were used for the evaluation of non-synonymous (Ka) and synonymous (Ks) values using TBtools (Chen et al., 2020). Tandem and collinear gene pairs inside the durum wheat genome were further used to evaluate both Ka and Ks using TBtools.

### 2.4 Phylogenetic analyses

Phylogenetic trees including *Arabidopsis thaliana* and *Oryza sativa* NPF and NRT2 genes and those here identified on durum wheat were constructed. The final dataset included 357 and 31 protein sequences for NPF and NRT2 families, respectively. Alignment was performed with the online tool CLUSTALW (Sievers et al., 2011) with default parameters. The unrooted phylogenetic tree was generated through the IQ-TREE software v. 2.2 (Nguyen et al., 2015) with the maximum likelihood method, 1000 bootstrap replicates, and the JTT + G4 model for both NPF and NRT2 trees, selected by the IQ-TREE best-fit model selection. Gene trees were visualized and analyzed through FigTree v. 1.4.4 (http://tree.bio.ed.ac.uk/software/figtree/).

### 2.5 Expression profiles of TdNPF and TdNRT2 genes and co-expression analysis

A total of 195 wheat RNA-Seq datasets were downloaded from the Sequence Read Archive (SRA). These included 13 durum cultivars, 9 tissues, and 25 phenological stages (Zadoks scale: from Z12 to Z90) (**Table S1**). Raw reads were trimmed with the Trimmomatic tool (Bolger et al., 2014) using the options: LEADING:3 TRAILING:3 SLIDINGWINDOW:4:20 MINLEN:50. Clean reads were then quantified using Salmon (Patro et al., 2017) with default parameters and normalized through DESeq2 (Love et al., 2014). Reads were further filtered using the SVA package (Leek et al., 2012) to remove any batch effect or unwanted sources of variation using 10 surrogate variables. A co-expression network analysis was carried out by using the Weighted Gene Co-Expression Analysis (WGCNA) method (Langfelder and Horvath, 2008) with the following parameters: soft threshold=12, minimum module size=100, mergeCutHeight=0.3. Co-expression networks for each module were analyzed using Cytoscape (Shannon, 2003) and the hub genes for each network were selected using the CytoHubba pluging (Chin et al., 2014). Furthermore, module-trait (conditions) relationship was evaluated as correlation between the eigengenes of each module and a binary matrix representing each condition. Heatmaps were generated using the Pheatmap R package (Kolde and Kolde, 2018) using log-transformed normalized counts.

### 2.6 Data retrieval

The sequences and annotation files of all genomes were downloaded from the Ensembl plants database (http://plants.ensembl.org) (Bolser et al., 2016). The *Aegilops speltoides Tausch*. genome was obtained from the e!DAL - Plant Genomics & Phenomics Research Data Repository (https://doi.org/10.5447/ipk/2022/0) (Avni et al., 2022). The RNA-Seq datasets used for the expression profile and the co-expression analyses were obtained from the SRA archive (Leinonen et al., 2010) (**Table S1**).

## 3 Results

### 3.1 Durum wheat NPF and NRT2 genes identification

To identify NRT2 and NPF genes in durum wheat, a BLASTP search against all predicted protein sequences of the genome using the full-length amino acid sequences from five different plant species was carried out. The output of the BLAST search was further scanned using the HMMER3 tool with the ‘Proton-dependent oligopeptide transporter family’ profile (IPR000109) and the PLN00028 NCBI domain, and finally 211 and 20 NPF and NRT2 genes, respectively in the durum wheat genome were identified. *NPFs* and *NRT2s* showed 103 and 6 homologous groups, respectively, between A and B genomes.

The *TdNPFs* showed high variability in both gene length and amino acids content. The nucleotide sequences of the 211 genes showed a 3400 bp average gene length and encoded proteins ranging from 71 to 943 amino acids, with an average length of 583 amino acids, and molecular weights ranging from 7 to 105 kDa.Eighty percent of the TdNPF proteins showed 12 predicted transmembrane domains, while almost 95% of these proteins were localized in the plasma membrane (**Figure S1**). Like in durum wheat, NRT2 is a smaller gene family with a lower variability compared to the NPF family. The twenty TdNRT2 members showed a 1600 bp average gene length and encoded proteins ranging from 113 to 573 amino acids, with a mean length of 509 amino acids. Their molecular weights ranged from 12 to 62 kDa. Seventy-five percent of the TdNRT2 proteins showed 12 predicted transmembrane domains, while 90% were predicted to be localized in the plasma membrane (**Table S2**).

### 3.2 *TdNPFs* and *TdNRT2s* phylogenetic analysis

**Figure 1:**
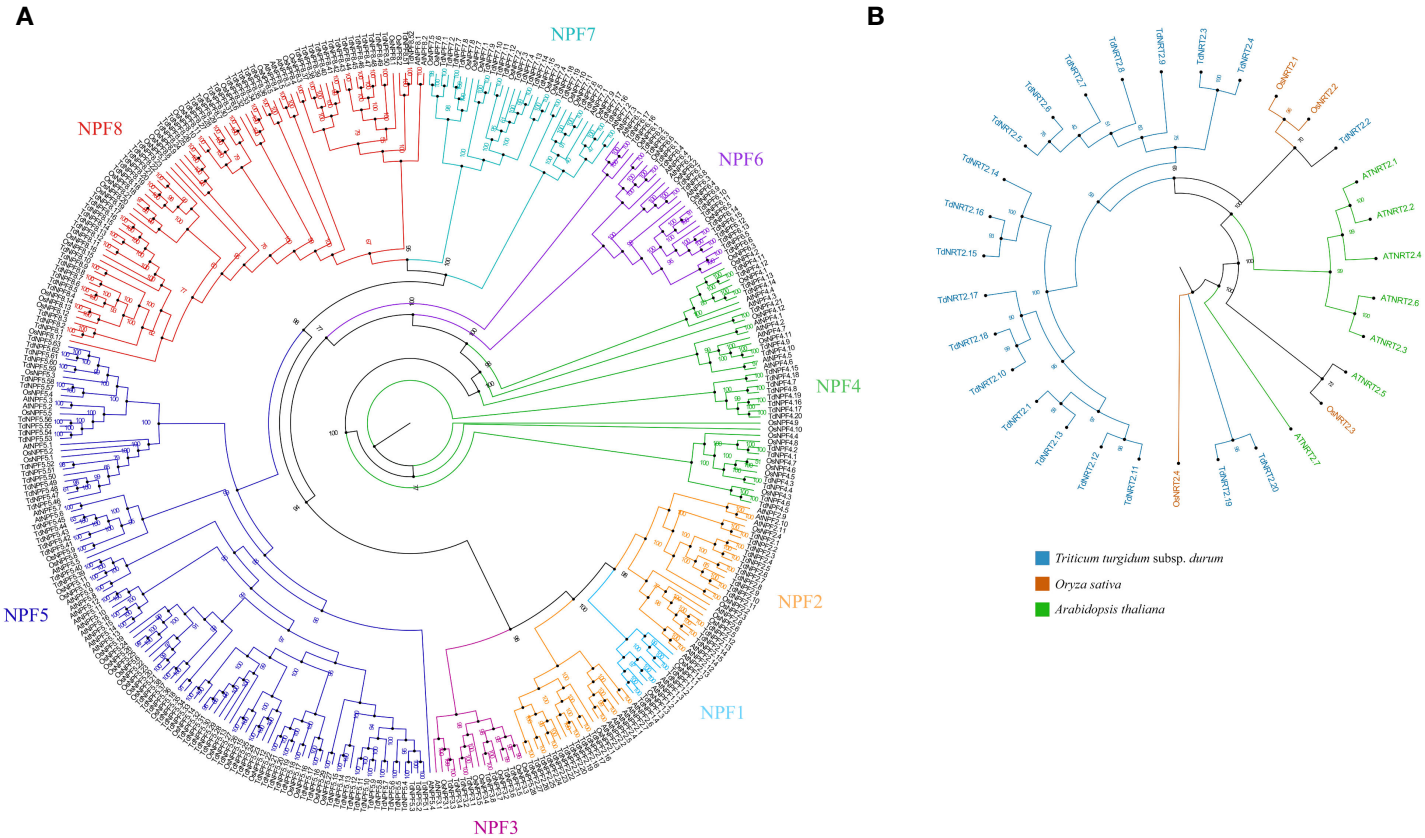
Phylogenetic analysis of the two gene families from *Arabidopsis thaliana*, *Oryza sativa* and *Triticum durum*. **(A)** Maximum likelihood tree of the NPFs full length protein sequences. The eight NPF sub-families are highlighted using colors: 1:Turquoise – 2:Orange – 3:Pink – 4:Green – 5:Blue – 6:Violet – 7:Cyan – 8:Red. **(B)** Maximum likelihood tree of the NRT2s full length protein sequences. The three species were highlighted using colors as indicated in the figure legend. Species specific branches were further highlighted using the same colors. Both trees were constructed using iqtree2.2, visualized, and modified using Figtree. Bootstrap values (1000 replicates) are shown for each node.

To explore the molecular evolution and the TdNPF gene family organization, we performed a phylogenetic analysis including protein sequences from *Arabidopsis thaliana* (53 *AtNPFs)*, *Oryza sativa* (93 *OsNPFs)*, and the 211 *TdNPFs* here identified in *Triticum durum* for a total of 357 NPF sequences. The Multiple Sequence Alignment (MSA) performed by CLUSTALW was used as input to IQ-TREE for both the model selection and the maximum-likelihood tree estimation. The phylogenetic tree showed a distinct clustering among the eight known NPF sub-families (**Figure 1A**). All the key nodes between sub-families are well supported with bootstrap values > 98 and all the genes from *Arabidopsis* and rice belonging to the same sub-family clustered together (**Figure 1B**). These results ensured the accuracy and reliability of the tree construction, suggesting a higher sequence variability between sub-families compared to the interspecific variability of each sub-family. The *TdNPFs* were assigned to the eight sub-families, namely from TdNPF1 to TdNPF8, following the tree topology and the previous classifications from other species. The sub-families TdNPF5 and TdNPF8 included the highest numbers of members (63 and 52, respectively), while TdNPF1 and TdNPF3 were the smaller sub-families with four and 8 genes, respectively (**Table S2**). TdNPF4, TdNPF5 and TdNPF6 were the only monophyletic groups, while the sub-families TdNPF1, TdNPF2, TdNPF3 and TdNPF7, TdNPF8 formed two distinct clusters with TdNPF1 clustering inside the TdNPF2 branch.

The NRT2 gene family was analyzed by using a similar approach; the maximum-likelihood phylogenetic tree was constructed based on the 31 NRT2 protein sequences, of which 7, 4 and 20 from *Arabidopsis*, rice and durum wheat, respectively. The phylogenetic tree revealed distinct evolutionary relationships among the NRT2 proteins of durum wheat and the other two species. Specifically, almost all the durum wheat NRT2 proteins formed a separate cluster, with only one protein (TdNRT2.2) closely grouped with the NRT2 proteins of *Oryza sativa* (OsNRT2.1 and 2.2) (**Figure 1B**). More interestingly, two other proteins (TdNRT2.19 and TdNRT2.20) showed a more ancient evolutionary divergence compared to all other TdNRT2 proteins, forming adistinct basal cluster, while all the AtNRT2 proteins clustered in three sub-clusters.

### 3.3 Chromosome location

The *TdNPFs* were evenly distributed along chromosomes in the A and B genomes (**Figure S2A**). The 2B chromosomal region (R2B) showed the highest gene density while the central chromosomal regions showed a lower gene density on average. Interestingly, four *NPFs*-enriched regions in chromosomes 2B, 3A, 3B, and 4B, also located in the R2B, were found, ranging from 5 to 7 genes per window (6Mb).

By contrast, the *TdNRT2s* were unevenly distributed along the genome, with chromosome 6 in both genomes (A and B) significantly enriched with 9 and 8 genes in 6B and 6A chromosomes, respectively (**Figure S2B**). Interestingly, all the *TdNRT2s* on chromosome 6 were located in the R1 in a significantly enriched window in both genomes (A and B) while only two gene members were found in the R2B (**Figure S2A**).

### 3.4 Gene structure and conserved motifs prediction

The gene structure of both gene families showed a significant difference in the number of exons and transcript isoforms (**Figure S3**). Most of the 211 *TdNPFs* exhibited more than two exons, with 85% of genes ranging from 3 to 6. The distribution of the transcript isoforms number was significantly different compared to the genome, with an average number of 3 transcript isoforms per gene. The *TdNRT2* gene family showed a lower number of exons (9 out of 20 genes with one exon) and a lower number of transcripts per gene with more than 50% of genes showing only one transcript isoform.

To highlight conserved motifs and analyze their distribution among sub-families, protein motif analysis was carried out using the MEME tool. All the TdNPF proteins showed highly conserved motifs patterns (**Figure S4**). Despite that, the spatial organization and distance between conserved motifs were highly variable. The intra-motif variability was very high, with only few positions conserved in almost all the protein sequences (**Figure S5**).

Functional characterization of these motifs was performed using the NCBI protein domain search using the most represented sequence for each of the 25 conserved motifs. 14 motifs were assigned to the Major Facilitator Superfamily (MFS) while the remaining (11) were not assigned to any known protein domain. The motif#1 was identified in all the 211 NPF proteins, while the less conserved motif#19 and motif#21 were found only in 98 and 99 proteins, respectively. Furthermore, motif#18 (FILGN**EFFER**LAYYG), shared by 147 TdNPF proteins, contains the highly conserved ExxER/K peptide, suggesting its involvement in proton binding and transport. Among these sequences, both glutamic acids (E) were conserved in 80% of sequences, while the arginine residue (R) is less conserved. Rare motif variants such as ExxDR and ExxEE were also detected.

15 conserved motifs were identified in the TdNRT2 gene family (**Figure S6**). Nine out of 15 were assigned to the NO_3_^-^ transmembrane transporter superfamily (PLN00028). Moreover, the distribution and position of the motifs created regular patterns and showed lower sequence variability compared to TdNPF family. Almost all the NRT2 genes shared many of the conserved motifs, except four highly variable genes (TRITD7Av1G231010, TRITD7Bv1G180680, TRITD2Av1G017380, TRITD6Bv1G008700).

### 3.5 Transcription factor binding sites, and CRE*s* prediction

Transcription Factors (TFs) are essential for modulating gene transcription levels and many TFs directly regulate the expression of NPF and NRT2 genes (Marchive et al., 2013; Liu et al., 2017). We predicted the TF binding sites in promoter regions (3,000 bp upstream of transcription start site) of TdNPFs and TdNRT2 using the Binding Site Prediction tool of the PlantTFDB, and more than four thousand (4,072) binding sites for 163 TFs were identified in the promoter regions of 197 *TdNPFs*. The most abundant families of TFs were MYB, AP2, and NAC (**Figure S7**). One hundred twenty (120) binding sites for 53 TFs were detected in the promoter region of 19 *TdNRT2s*, of which the AP2 family resulted the most abundant. Interestingly, almost 96% (51 out of 53) of the TFs families were shared between NPF and NRT2 genes promoter region.

Cis-regulatory elements (CRE) are non-coding DNA regions also involved in the transcription regulation of neighboring genes (Bai et al., 2013). Here, we predicted CREs in the promoter regions of both *TdNPFs* and *TdNRT2s* using PlantCARE. Five thousand one hundred and twenty-one (5,121) CREs of 27 different types in the 211 promoter regions of TdNPF were found (**Figure S7**). The most abundant sites were the ABA responsive element (ABRE), DRE and MYB binding sites, activation sequence-1 (as-1), and the stress response element STRE, accounting for 70% of all the CREs. Other less abundant CREs were involved in light-response (G-box), biotic and abiotic stress response (MYC), and the common TATA-box and CAAT-box. One thousand five hundred and eighteen (1,518) CREs were predicted in the promoter regions of *TdNRT2s*. They were highly enriched in MYB and MYC binding sites, with many genes showing more than 5 sites in their upstream sequence accounting for almost 40% of CREs, in agreement with the previously described TF binding site prediction.

### 3.6 Expression profiles and co-expression analysis

**Figure 2:**
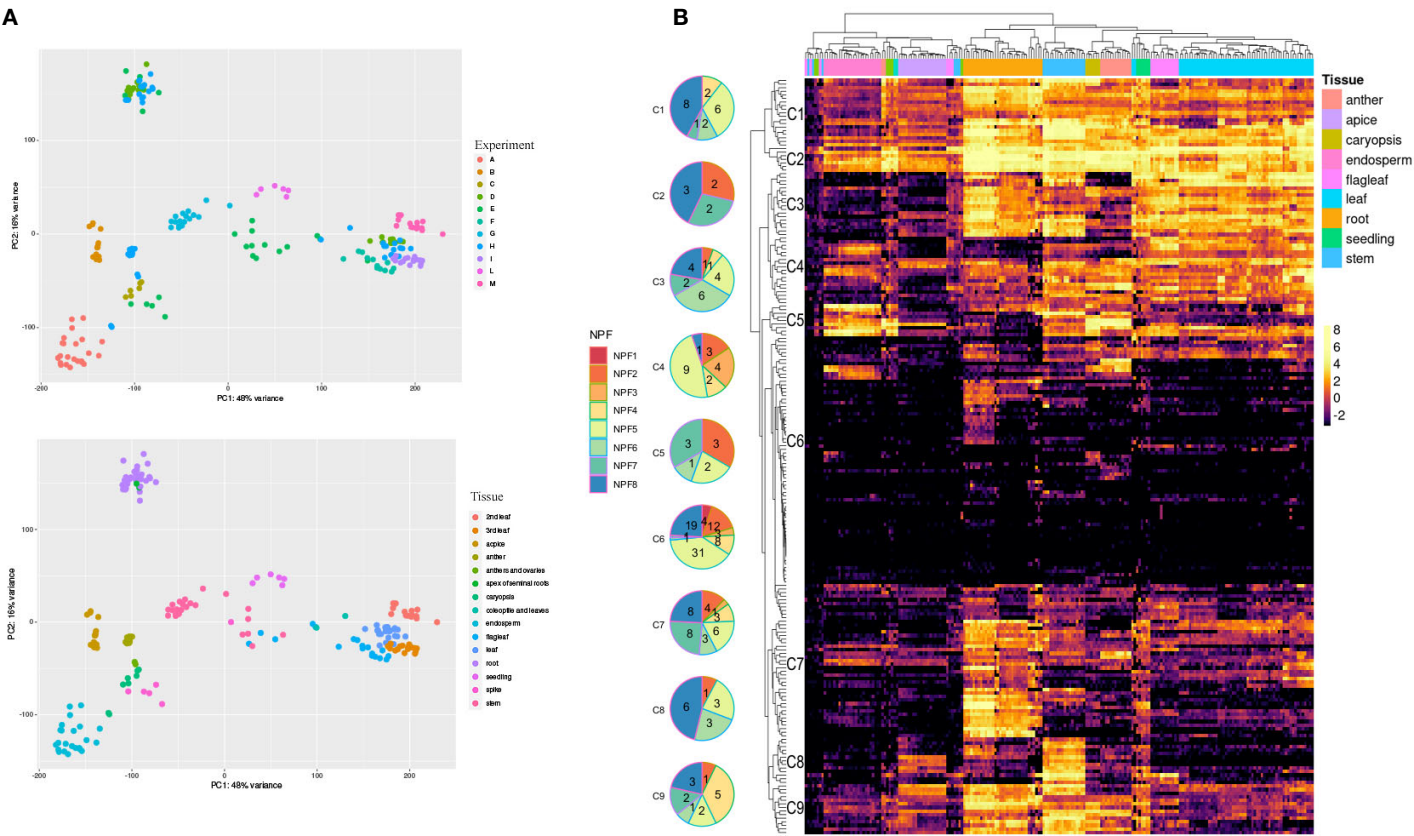
RNA-Seq of 195 samples obtained from the Short Reads Archive (SRA). **(A)** Principal Component Analysis performed with DESeq2 after the SVA correction. We highlighted SRA experiments (Top) and tissues (bottom). **(B)** Expression profiles of TdNPF genes in 9 tissues. Hierarchical clustering was performed both on rows and columns. Rows were roughly divided into 9 groups according to the similar expression levels. Pie charts were used to highlight the abundance of each NPF sub-family in each cluster. Tissues were highlighted as colored bars at the top of the heatmap.

The expression profiles of 211 TdNPF and 20 TdNRT2 genes were detected using publicly available RNA-seq datasets from the Sequence Reads Archive (SRA) covering 9 tissues at different growth stages from 13 different cultivars (**Figure 2A**). The hierarchical clustering based on TdNPF genes showed a clear tissue-specific signal, with almost all the samples from the same tissue clustering together (**Figure 2B**). The 211 NPF genes were further divided roughly into 9 clusters based on their expression patterns. These clusters ranged from 7 to 79 genes, with an average of 18 genes. Almost all the clusters showed the highest expression in roots, stem, and leaf, with five clusters being the most expressed (Cluster1-5). Interestingly, the cluster with the higher number of genes (Cluster 6) showed very constant low gene expression levels in almost all samples, except two small groups of genes induced in anthers, endosperm and roots.

TdNRT2 showed an opposite trend compared to TdNPF, with limited correlation between gene expression and tissue, except in the roots and seedlings (**Figure S8**). As might be expected, the higher gene expression was detected in roots, with 7 NRT2 members, highly similar to *AtNRT2.1* and *AtNRT2.4*, that showed higher expression (TdNRT2.3,.4,.5,.6,.7,.8,.9) (**Figure S8**).

**Figure 3:**
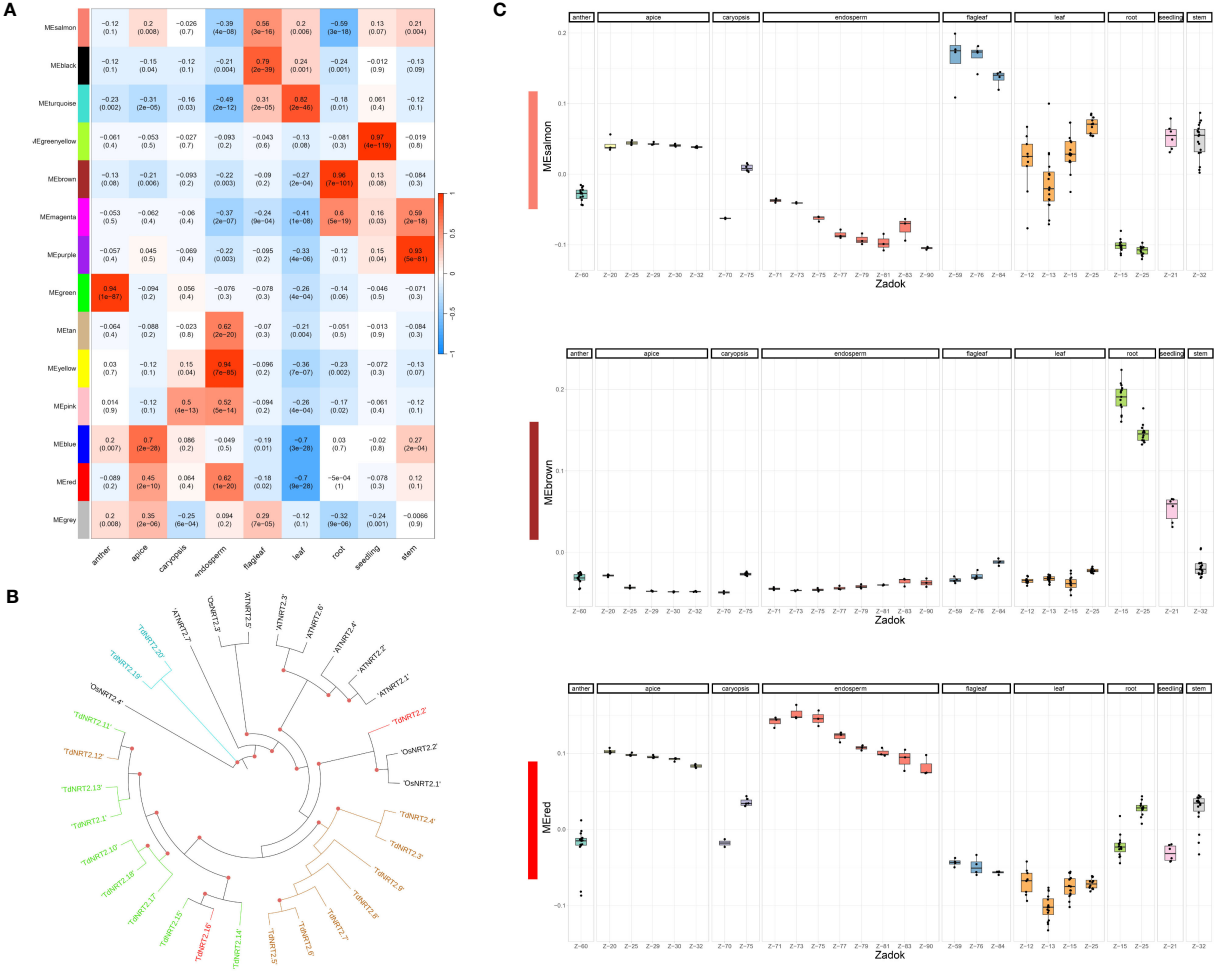
Weighted Gene Co-expression Network Analysis (WGCNA). **(A)** Heatmap of the Correlation coefficients between Module Eigengenes (WGCNA) and tissues. Pearson Correlation coefficient was evaluated using a binary matrix representing each tissue (1) against all the others (0). **(B)** Phylogenetic tree of TdNRT2 genes. Genes within each co-expression module form distinct clusters on the tree, visually distinguished by the use of module-specific colors (turquoise, red, brown and green). **(C)** Expression profile of the three most abundant co-expression modules visualized using the Boxplots of Module Eigengenes. Each tissue was highlighted in facets, while the developmental stage (Zadok) was highlighted on the X-axis.

The same dataset was used to evaluate the co-expression of both NRT2 and NPF gene families adopting the Weighted Gene Co-expression Network Analysis (WGCNA) method. We detected fourteen co-expression modules which showed highly variable expression profiles. 87.2% of durum wheat genes were assigned to co-expression modules, with four modules, colored salmon, red, blue and brown, showing a significantly higher number of genes ranging from roughly thirteen thousand to eight thousand. Module-tissue relationships were evaluated to highlight each module expression profile (**Figure 3A**). TdNRT2s were assigned to modules brown (8), green (8), red (2) and turquoise (2) which were highly induced in roots, anthers, endosperm-apex and leaves-flag leaves, respectively (**Table S7**). These expression profiles closely correlated to the phylogenetic tree distribution of the NRT2 genes, with almost all *TdNRT2* in the same co-expression module clustering together (**Figure 3B**). Furthermore, all six NAR2/NRT3 genes in the durum wheat genome were assigned to the brown module, highlighting their combined action mainly in roots.

TdNPFs showed a wider range of expression patterns, in agreement with the hierarchical clustering. They were assigned to ten of the fourteen modules detected. The majority of NPF genes belonged to the brown (52) and salmon (48) modules which were induced in root and flag leaf, respectively (**Figure 3C**). Interestingly 35 TdNPFs were assigned to three modules, red (23), yellow (9) and pink (3), highly upregulated in the caryopsis, especially in the endosperm, with three slightly different expression profiles (**Figure S9**). Furthermore, the network analysis allowed us to detect the hub-genes in each module. Among these we detected three NPF genes, *TdNPF6.12*, *TdNPF6.8* and *TdNPF5.61*, belonging to brown, red and salmon modules, respectively. We further used co-expression modules to detect expression patterns in homologous genes among the two genomes (A and B). The half (50.4%) of the TdNPFs homologous belonged to different co-expression modules while only two NRT2 genes did not cluster in the same module.

### 3.7 NPF gene sequence divergence and collinearity in five species of the Triticeae tribe

**Figure 4:**
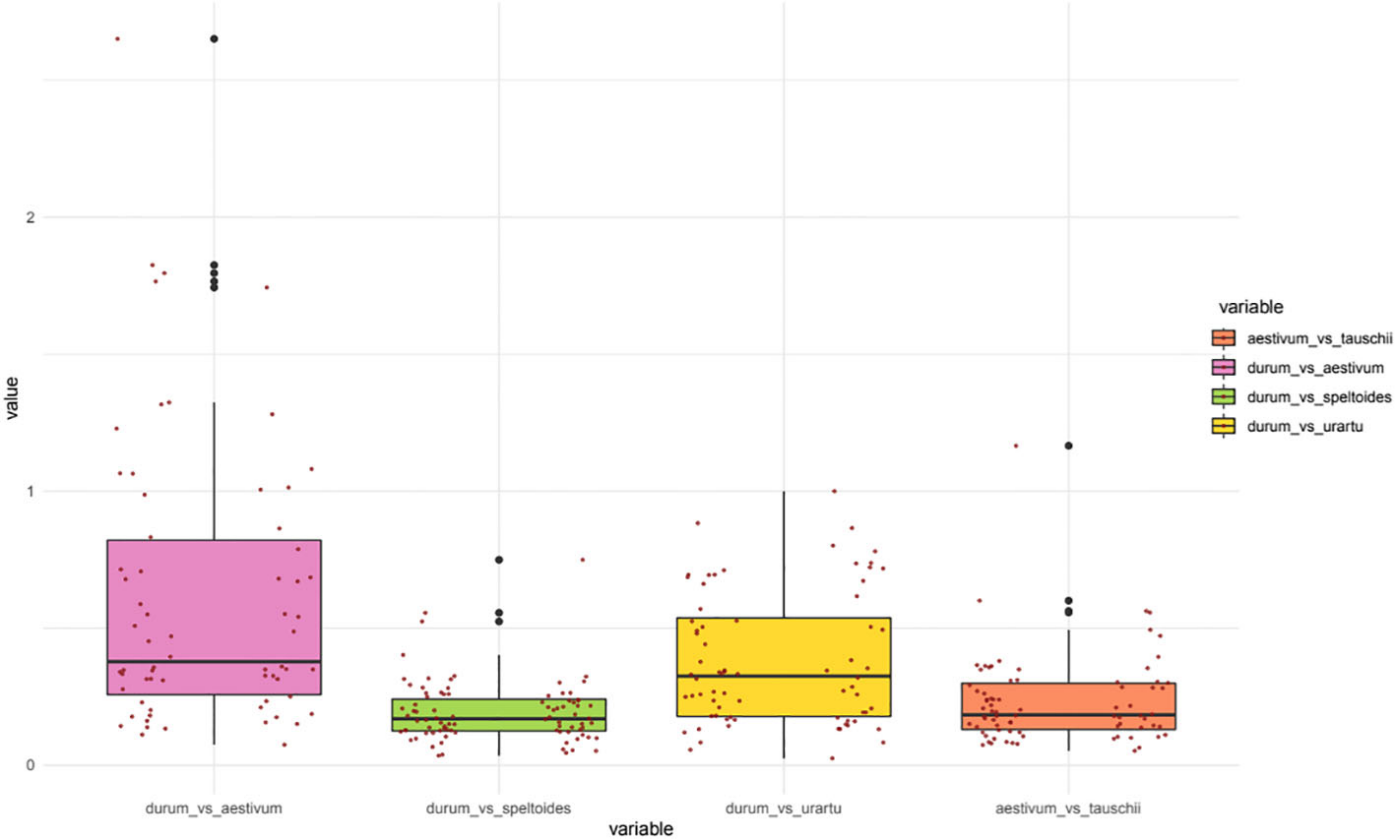
Boxplots of the Ka/Ks values for the orthologous NPF genes between five poaceae species: Durum wheat (*Triticum turgidum* subsp. *durum*), Bread wheat (*Triticum aestivum*), *Aegilops speltoides* Tausch, *Triticum urartu* and *Aegilops tauschii*. Two scatter plots (left: TdNPF on the A sub-genome; right: TdNPF on the B sub-genome) highlights the single Ka/Ks for each gene pairs.

Evolutionary constraints of durum wheat NPF and NRT2 genes was evaluated through pairwise comparisons of Ka/Ks values from five species belonging to the Triticeae tribe (**Figure 4**). In detail, the durum wheat *TdNPFs* were compared to their orthologs in *T. urartu*, *Ae. speltoides, Ae. tauschii* and *T. aestivum* genomes. Furthermore, the Ka/Ks values of each duplicated TdNPF gene pairs were also evaluated. Ka/Ks was evaluated for 170, 91, 83, 74 orthologs in the *durum*/*aestivum*, *durum*/*speltoides*, *durum*/*urartu* and *aestivum*/*tauschii* comparisons, respectively. Interestingly, both the *durum*/speltoides and the *aestivum*/*tauschii* comparison showed very low Ka/Ks values with an average of 0.19 and 0.23, respectively, by contrast, the highest values were detected in the *durum*/*aestivum* comparison with an average of 0.49. Five genes (TdNPF3.5, TdNPF4.7, TdNPF5.42, TdNPF7.12, TdNPF8.38) exhibited Ka/Ks values greater than 1.5, indicating a substantial positive selection acting on these genes. On the other hand, ten genes displayed a Ka/Ks value close to 1, suggesting a relatively neutral selection. Among the five genes with Ka/Ks > 1.5, three were associated with the brown co-expression module, indicating their upregulation specifically in roots. Based on these results, the significant difference between *durum*/*aestivum* and the other three comparisons was confirmed by Tukey’s test (**Figure S10**).

Ka/Ks was evaluated also on durum wheat NPF and NRT2 tandem duplicated genes and NPF collinear genes between the two sub-genomes. All the gene pairs comparisons showed Ka/Ks values lower than 1, rarely higher than 0.5, suggesting strong purifying selection acting on duplicated genes, regardless the duplication event type. In particular, tandem duplications showed a slightly higher Ka/Ks among the NPF genes with an average of 0.31 compared to collinear duplicated NPF genes between sub-genome A and B (average 0.26). Finally, NRT2 showed a drastically lower Ka/Ks value ranging from 0.1 to 0.01.

**Figure 5:**
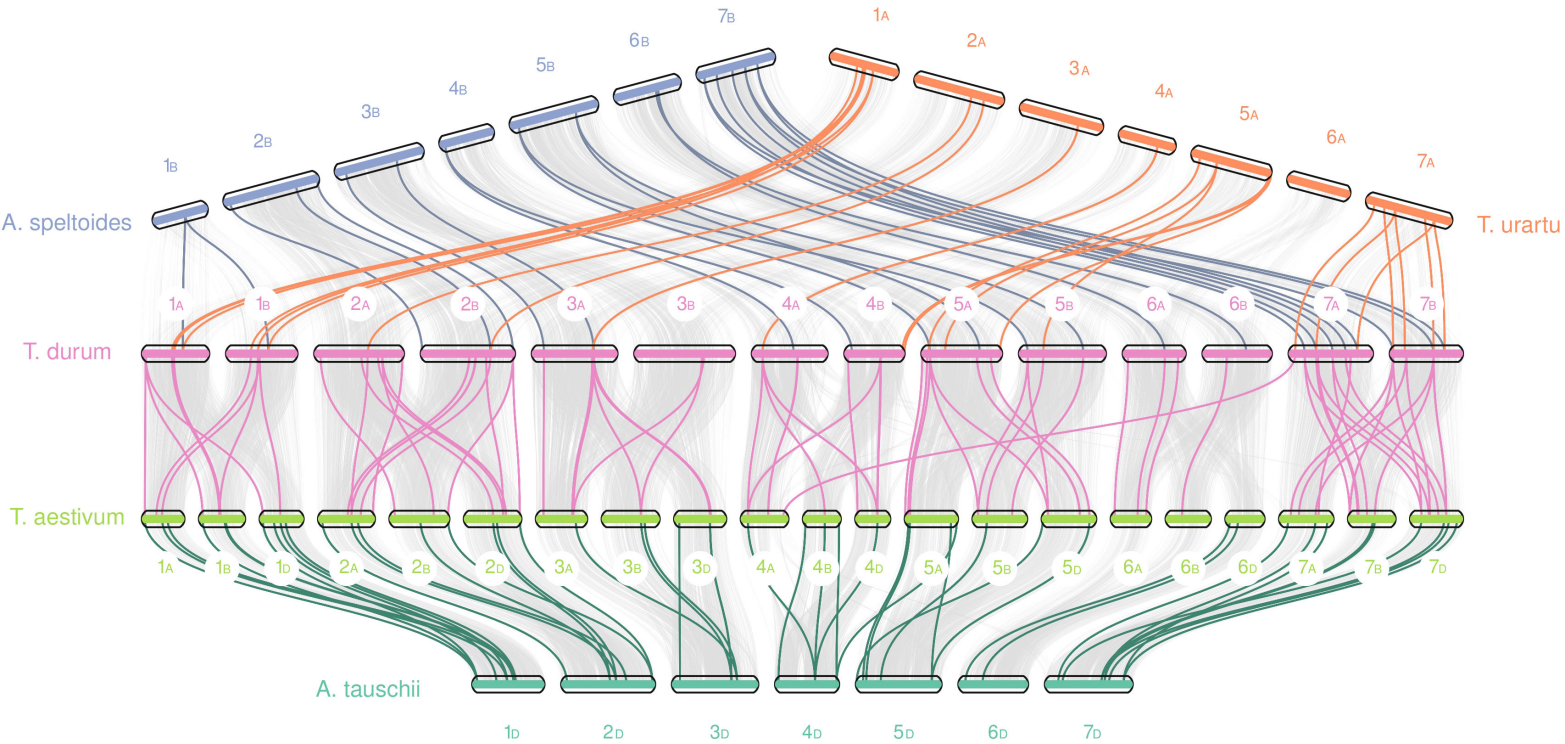
Collinearity analysis of NPF genes between five species from the Trititceae tribe. Grey lines indicate collinear blocks between genomes, while the colored lines indicate NPF genes detected inside collinear blocks. Sub-genomes were highlighted using the A, B or D letters.

Furthermore, using collinearity analysis through MCScanX we were able to characterize the relationships and the duplication events of both gene families inside the durum wheat genome and between these five species (**Figure 5**). In the durum wheat genome, almost 45% of *TdNPFs* were included in collinear pairs detected between A and B genomes. In detail, 77 segmental and 94 tandem duplications, as well as fewer dispersed (30) and proximal duplication (10) were detected; 42% of *TdNPFs* formed tandem blocks, with 11 blocks including three or more genes.

*TdNRT2s* are mainly located in two enriched regions on chromosomes 6A and 6B, as previously highlighted. These formed 5 tandem blocks, 3 and 2 located on 6B and 6A chromosomes, respectively. Furthermore, 14 tandem and no segmental duplications were detected.

Interspecific analysis of NPF genes revealed 23 and 25 collinear blocks in durum-speltoides and durum-urartu comparisons, respectively, 48 and 75 pairs in aestivum-aegilops and durum-aestivum comparisons, respectively. Almost all the blocks were detected between homologous chromosomes among the five genomes, significant differences in the number of blocks between A, B or D sub-genomes were not detected.

## 4 Discussion

In plants, both the NPF and the NRT2 gene families are involved in nitrate/nitrite uptake, translocation and remobilization. *NPFs* are also involved in the transport of many other substrates such as hormones, secondary metabolites, peptides, chloride and potassium. A deeper characterization of these gene families is crucial to understand plant nitrate and metabolite transport.

In the present study, 211 *TdNPFs* and 20 *TdNRT2s* were identified in the *Triticum turgidum* L. subsp*. durum* (Desf.) Husn. genome. These numbers were comparable to other allopolyploid species such as *Brassica napus* (199 NPFs, 17 NRT2s), *Saccharum spontaneum* (178, 20), and *Triticum aestivum* (331, 46) and significantly higher than many diploid monocots and dicots such as *Arabidopsis thaliana* (53 and 7), *Oryza sativa* (93, 4) and *Zea mays* (79, 1). The NPF gene family expansion in plants seems to have arisen from neo- and sub-functionalization, as suggested by many reports (Lynch and Conery, 2000; O’Brien et al., 2016; Jørgensen et al., 2017; Wang et al., 2019a). In wheat, the large number of members in both gene families could be involved in highly differentiated responses to the availability of various substrates. Indeed, the high number of TdNPF and TdNRT2 genes, deriving from recent polyploidization and duplication events, may provide a higher modularity in terms of substrate affinity, condition or tissue specific gene expression induction and new protein-protein interactions. Similar effects were reported in many allopolyploid species such as rice, soybean, cotton and, in the MIKC-type MADS-box gene group, in bread wheat (Flagel et al., 2008; Schilling et al., 2020; Lee and Szymanski, 2021). In *Triticum aestivum*, the diversification of both NPF and NRT2 gene families was likely due to drift, leading to significant differences in the N-use efficiency of subpopulations clustered based on Single Nucleotide Polymorphisms (SNPs) within the NPF and NRT2 genes (Li et al., 2021).

Our phylogenetic analysis highlighted a high divergence among NRT2 genes from durum wheat, rice and *Arabidopsis* with genes from each species clustering together in distinguished groups. The best hit (BLASTP) of many *TdNRT2s* against the *Arabidopsis* NRT2 protein sequences is often *AtNRT2.1*, making it hard to functionally characterize these transporters solely based on their sequence homology, thus the association of previous functional annotations from model species to the newly identified genes in durum wheat become challenging. Here, we focused on sequence, gene expression and protein domains characterization, but further studies will be needed to fully describe *TdNRT2s* at a functional level.

The phylogenetic analysis on the NPF genes yielded more informative results, with all the orthologous genes belonging to the same sub-family clustering together, allowing us a much more reliable annotation of the novel *TdNPFs*. These results support the hypothesis of NPF family divergence before the separation of monocots and dicots, as suggested by Wang et al. (2019b). Furthermore, the NPF1 branch clustered inside the NPF2 branch, breaking it in two sub-classes. This feature was observed in other species such as *Brassica napus* and often led to the definition of more than eight sub-families, with NPF2 split into NPF2-1 and NPF2-2(Wen et al., 2020). Interestingly, this split was not detected in *Triticum aestivum* (Li et al., 2021; Kumar et al., 2022) potentially due to slightly different plant species utilized or to slightly different clustering method.

The chromosome location of both transporter families showed a non-random distribution inside the genome. Interestingly, *TdNRT2s* are highly concentrated on chromosomes 6 from both genomes (A and B), probably due to the multiple tandem duplication events, as shown by the results of collinearity analysis. In *Arabidopsis*, the *AtNRT2.1* and *AtNRT2.2* genes are adjacent, and end to end on chromosome 1, and this apparent duplication has been seen for orthologues in other species. AtNRT2.5 is also located on chromosome 1. Three other NRT2s are located on chromosome 5, with only AtNR2.6 located on chromosome 3. A similar enrichment on chromosome 6 of all three genomes (A, B and D) was detected in bread wheat, also deriving from tandem duplication that was suggested to have arisen from unequal crossing-over events (Li et al., 2021). Although similar, the number of NRT2 genes in these genomic regions is higher in bread wheat, suggesting that some of these duplication events should have occurred after or during the hybridization of durum wheat with *Aegilops tauschii* (genome D) as supposed by the International Wheat Genome Sequencing Consortium (IWGSC) (2014). Further studies on the intraspecific variability of these gene families among the main wheat species could help to deeply understand how their expansions occurred and what type of mechanisms underlie their preservation after duplication.

In *Arabidopsis*, several putative NO_3_^−^ response cis-regulatory elements (CREs) have been detected in many promoters of N-related genes, while limited information is available for other plant species (Konishi and Yanagisawa, 2010; Wang et al., 2010; Rolly and Yun, 2021). Here, a high number of CREs related to ABA signaling and binding of Drought Responsive Element (DRE) and MYB TFs were detected in the *TdNPFs* promoter regions. Interestingly, the *TdNRT2s* upstream region was also highly enriched with MYB binding sites, as shown by both CREs and TF binding site prediction. MYB TFs are often involved in abiotic and biotic stress responses as well as in plant development, root and flower development (Kaur et al., 2017), although their role in NO_3_^–^related regulation has also been reported (Todd et al., 2004; Wang et al., 2018a; Zhang et al., 2021; Puccio et al., 2022). Interestingly, both gene family promoters showed multiple putative MYB binding sites for many genes. The presence of multiple binding sites for the same TF on the promoter of one or more genes has been often associated with a higher sensitivity to specific TFs (Howard and Davidson, 2004; Yáñez-Cuna et al., 2013; Brendolise et al., 2017).

The presence of many CREs and the partial overlap of their functions between the two gene families suggested that a complex regulatory network may be involved in modulating and fine-tuning their expression, with some TFs putatively involved in the regulation of members of both families. These could be involved in the spatiotemporal- or tissue-specific activation of transporter genes or may take part in the signaling cascade in response to the fluctuations of specific substrates concentration into the soil. Interestingly, the same analysis performed on the NPFs from *Brassica napus* yielded similar results on NPF genes (Wen et al., 2020), suggesting that the regulation of this gene family may involve the same TFs classes and could be evolutionarily conserved.

Fourteen motifs were assigned to the Major Facilitator Superfamily (MFS), the remaining 11 were not assigned to any known protein domain, suggesting a highly specific function for these peptides (putatively species-specific). Interestingly, 11 TdNPF7 proteins showed the monocot-specific variant ExxES, which is associated to non-proton dependent nitrate uptake and specific to the NPF7 sub-family (Longo et al., 2018). These genes were defined as NPF7a in rice, involved in the low-affinity nitrate transport system with some being tonoplast located (Hu et al., 2016).

Overall, *NPFs* showed a more variable gene structure and sequence variability in their conserved motifs compared to *NRT2s*. Interestingly, the *NRT2s* showed a simpler gene structure, with only one or two exons and highly clustered genomic locations. Both the gene structure and the chromosome locations of *NRT2s* seemed highly conserved among many monocots such as *Brachypodium distachyon*, *Saccharum spontaneum*, and bread wheat, mainly distributed on two chromosomes and harboring mainly one or two exons (Wang et al., 2019a; Li et al., 2021).

The high percentage of *NPFs* deriving from segmental and tandem duplication (37% and 44%, respectively) found in durum wheat supported the role of these genomic events in the expansion of NPF genes already reported in bread wheat (Li et al., 2021). Furthermore, the collinear analysis between durum and bread wheat and the putative A, B and D genomes was not able to detect a significantly higher number of collinear blocks between each putative sub-genome donor and the respective sub-genomes in durum or bread wheat. This observation does not directly support the idea that the expansion of these families derives from ancient duplication events in diploid wheat species, which should have occurred before hybridization into allopolyploid species (Salse et al., 2008). Instead, our results indirectly support the idea that the substantial increase in gene members in both these families is mainly due to tandem and segmental duplications in the tetraploid or hexaploid ancestral genomes, and not in the diploid ancestral genomes. These duplications seemed favored by polyploidization events, with bread wheat showing a higher number of duplication events (Buchner and Hawkesford, 2014; Kumar et al., 2022). Furthermore, tandem duplicated NPF genes in durum wheat genomes showed strong purifying selection, suggesting preserved function after duplication, in agreement with many studies on other gene families (Hu et al., 2018; Hajiahmadi et al., 2020; Zhu et al., 2020).

In allopolyploid species, gene expression patterns can be significantly altered and this is one of the main sources of phenotypic variation (Jackson and Chen, 2010). Here, by using 195 RNA-seq durum wheat datasets the expression profiles highlighted different trends in both gene families. The *TdNPF*s expression patterns resulted highly tissue-specific, with most samples from specific tissue forming distinct clusters. By contrast, NRT2 genes were predominantly expressed in roots and anthers, being assigned to brown and green modules. The distinctiveness of these two groups of *TdNRT2*s becomes even more evident, as we observed that all the NAR2/NRT3 genes are present in the brown module. This finding implies that most of the NRT2 genes in wheat are either engaged in root uptake, facilitated by NAR2/NRT3, or have undergone evolutionary adaptations for translocation or accumulation in anthers/seeds. Additionally, four members of *TdNRT2* showed a more complex expression profile, with *TdNRT2.2* and *TdNRT2.16* being highly induced in apex, grain and endosperm during maturation, while *TdNRT2.19* and *TdNRT2.20* being expressed in leaves and flag-leaves. The detection of NRT2 genes responsible for seed N-accumulation, such as *TdNRT2.2* and *TdNRT2.16*, could be crucial to increase yield and higher N content, as already demonstrated by their overexpression in bread wheat (Li et al., 2020). *TdNRT2.19* and *TdNRT2.20* were the most basal genes in our phylogenetic analysis together with *OsNRT2.4* and *AtNRT2.7* in agreement with recent reports (Li et al., 2021; Deng et al., 2023; Kumar et al., 2023). Interestingly, both *OsNRT2.4* and *AtNRT2.7* are mainly expressed in the tonoplast of maturing seeds and roots, which seems to suggest differentiated functions of these basal genes in the vacuole (Chopin et al., 2007; Wei et al., 2018) and contrasting with most other family members located in the plasma membrane.

*TdNRT2.2* was closely related to both *OsNRT2.1* and *OsNRT2.2*, which are usually expressed in root and germinating seeds (Feng et al., 2011). Furthermore, both phylogenetic and co-expression clustering yielded mostly the same results, with almost all the *TdNRT2s* from the same phylogenetic branch belonging to the same co-expression module. These results highlighted a close relationship between nucleotide variation and gene expression in this family, suggesting a shared selection mechanism between coding and regulatory regions. Similar coordinated evolution has been already observed in many gene families in mammals and plants (Necsulea and Kaessmann, 2014; Wang et al., 2020; Winkelmüller et al., 2021). Furthermore, duplication events may induce expression shifts favored by gene neo-functionalization as suggested by Fukushima and Pollock (2020), and this hypothesis could enhance the co-evolution of genome and transcriptome in the *NRT2* gene family in durum wheat.

The expression profiles of homologous genes showed significant variation, mainly within the *NPF* family. Indeed, about half of the NPF homologues exhibited dissimilar expression patterns. Although these differences may have already been present in ancestral genomes, the maintenance or development of highly similar genes with different expression patterns may provide a greater degree of modularity for regulation.

## 5 Conclusions

Our approach led to a comprehensive characterization of the NPF and NRT2 gene families in the durum wheat genome. Manual annotation of these transporters is crucial for understanding NO_3_^-^ and N dynamics and their impact on NUE in durum wheat. This study identified 211 TdNPF and 20 TdNRT2 genes for the first time, providing detailed insights into their protein sequences and conserved domains and on their regulatory elements. By extensively analyzing nearly all publicly available RNA-seq datasets, we achieved the most comprehensive characterization of both gene expression profiles and co-expression relationships. This investigation confirmed that a considerable number of these genes underwent neo- or sub-functionalization following small-scale duplication events. These findings indicate that the expansion of these gene families in wheat holds promising potential as a valuable resource for identifying NUE-related genes and as potential candidates for molecular markers and the development of transgenic plants. By understanding the key players involved in durum wheat production and incorporating these findings into future research, we can take significant steps towards more eco-friendly and sustainable durum wheat fertilization management, addressing a critical challenge in modern agriculture.

## Data availability statement

The original contributions presented in the study are included in the article/**Supplementary Material**. Further inquiries can be directed to the corresponding authors.

## Author contributions

GP: Conceptualization, Data curation, Formal Analysis, Investigation, Methodology, Software, Supervision, Validation, Visualization, Writing – original draft, Writing – review & editing. RI: Conceptualization, Supervision, Validation, Visualization, Writing – review & editing. DG: Conceptualization, Funding acquisition, Project administration, Resources, Supervision, Writing – review & editing. AF: Funding acquisition, Project administration, Resources, Supervision, Writing – review & editing. AH: Conceptualization, Formal Analysis, Investigation, Methodology, Supervision, Writing – review & editing. FS: Conceptualization, Formal Analysis, Investigation, Methodology, Resources, Supervision, Validation, Writing – review & editing. FM: Conceptualization, Formal Analysis, Funding acquisition, Investigation, Methodology, Project administration, Supervision, Validation, Visualization, Writing – review & editing.
