# Supplementary material for: Exploring the genetic landscape of nitrogen uptake in durum wheat: genome-wide characterization and expression profiling of NPF and NRT2 gene families
Source: Front Plant Sci. 2023 Nov 9;14:1302337. doi: 10.3389/fpls.2023.1302337 (PMC10665861; doi:10.3389/fpls.2023.1302337)
Supplement: Supplementary file 1 [file DataSheet_1.zip › Supplementary Figures.DOCX]

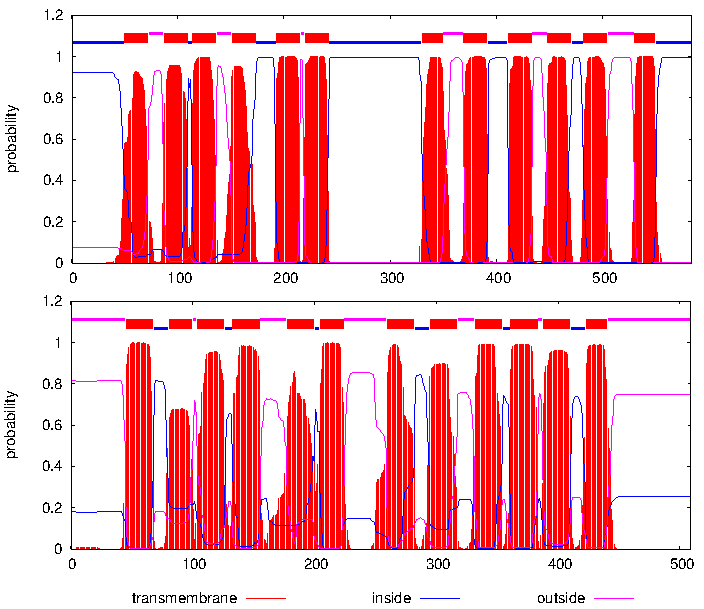
**Figure S1**. Prediction of transmembrane regions of TdNPF (top) and TdNRT2 (bottom) genes. The probability (y-axis) of each region being either transmembrane (red), outside (pink) or inside (blue) the plasma membrane.


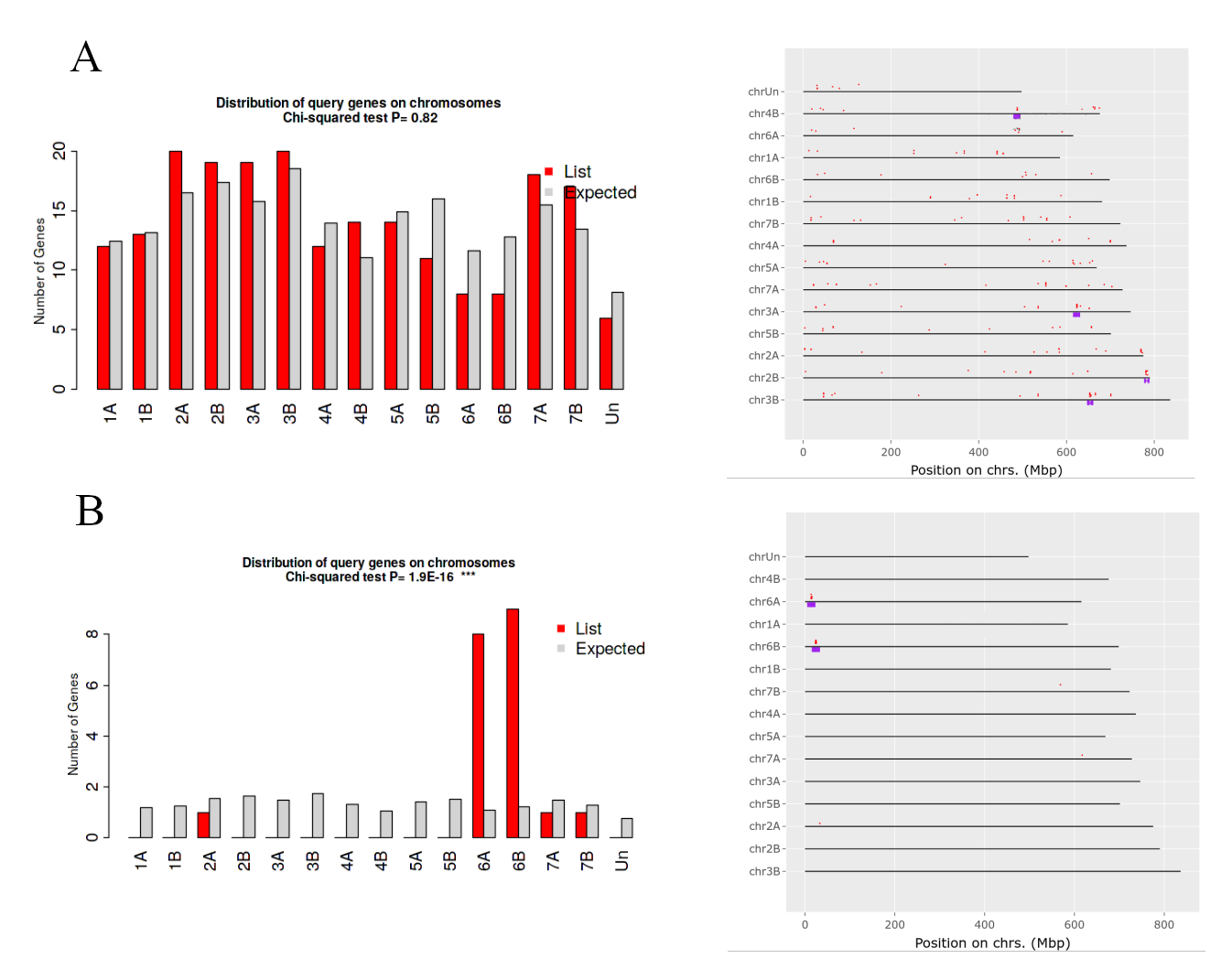
**Figure S2. Left:** Distribution of TdNPF (A) and TdNRT2 (B) genes on the 14 chromosomes of the durum wheat genome. **Right:** Hypergeometric test (FDR < 1e-05) on a sliding window scanning approach with a sliding window size set to 6MB for both TdNPF (A) and TdNRT2 (B) genes.


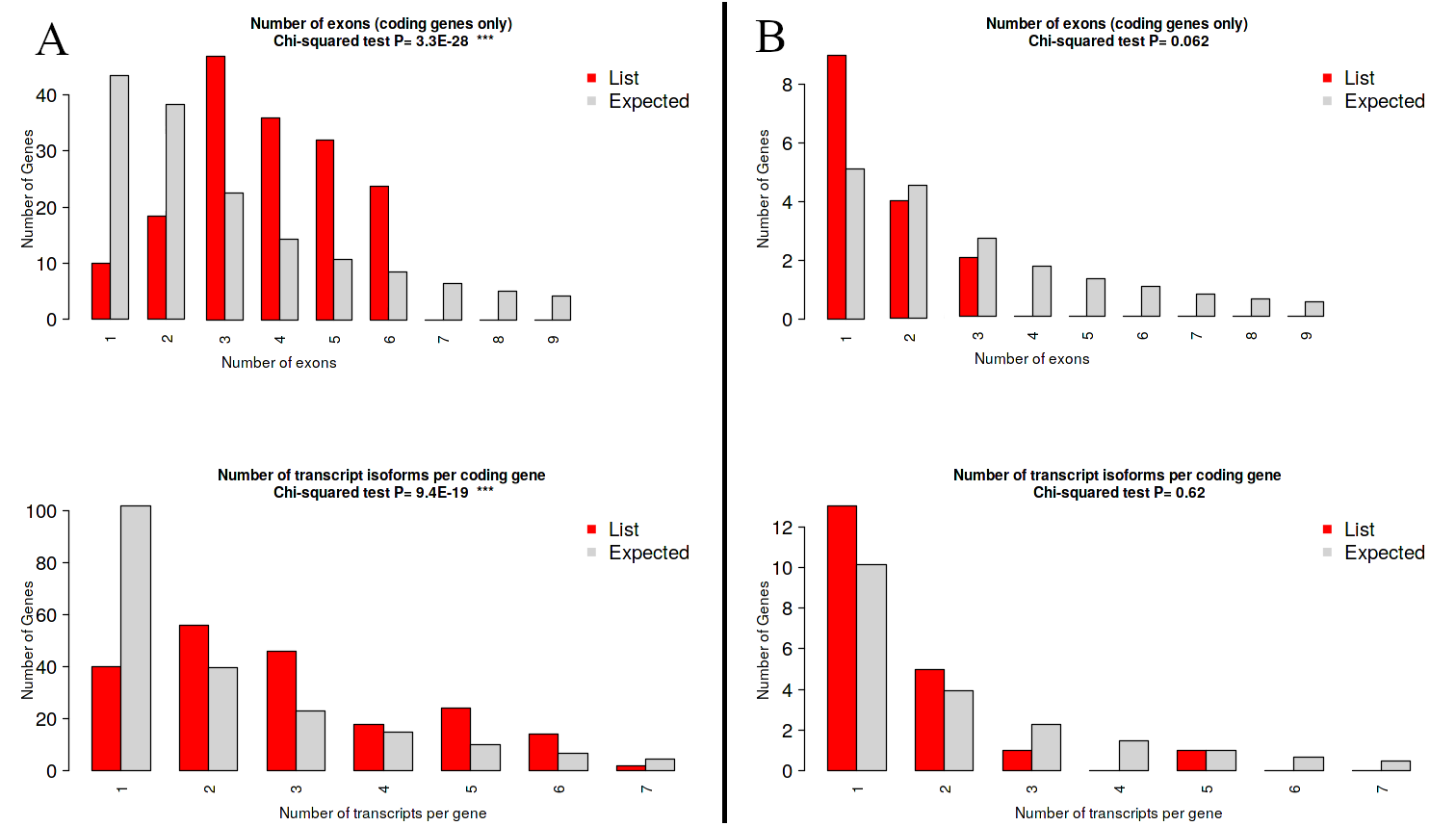
**Figure S3**. Number of exons (Top) and transcripts (bottom) of the 211 TdNPF (A) and TdNRT2 (B) genes. Chi-squared test was used to test if query genes significantly differ in terms of exons and transcripts abundance compared to all the background genes in the genome.





**Figure S4**. Conserved motifs detected in TdNPF protein sequences. The eight NPF sub-families are highlighted using colors: 1:Turquoise – 2:Orange – 3:Pink – 4:Green – 5:Blue – 6:Violet – 7:Cyan – 8:Red. The most represented motif for each of the 25 motifs is shown. The phylogenetic tree based only on TdNPF protein sequences is shown.


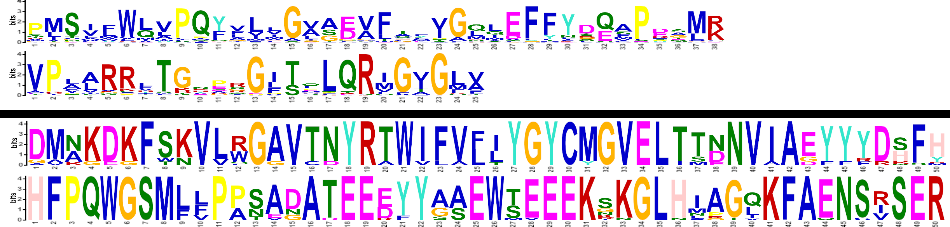


**Figure S5**. Sequence logos of the two most conserved motifs (Motif1 and Motif2) of both TdNPF and TdNRT2 identified using the MEME tool. The dimension of the letters represents the position-specific degree of conservation of the amino acid.


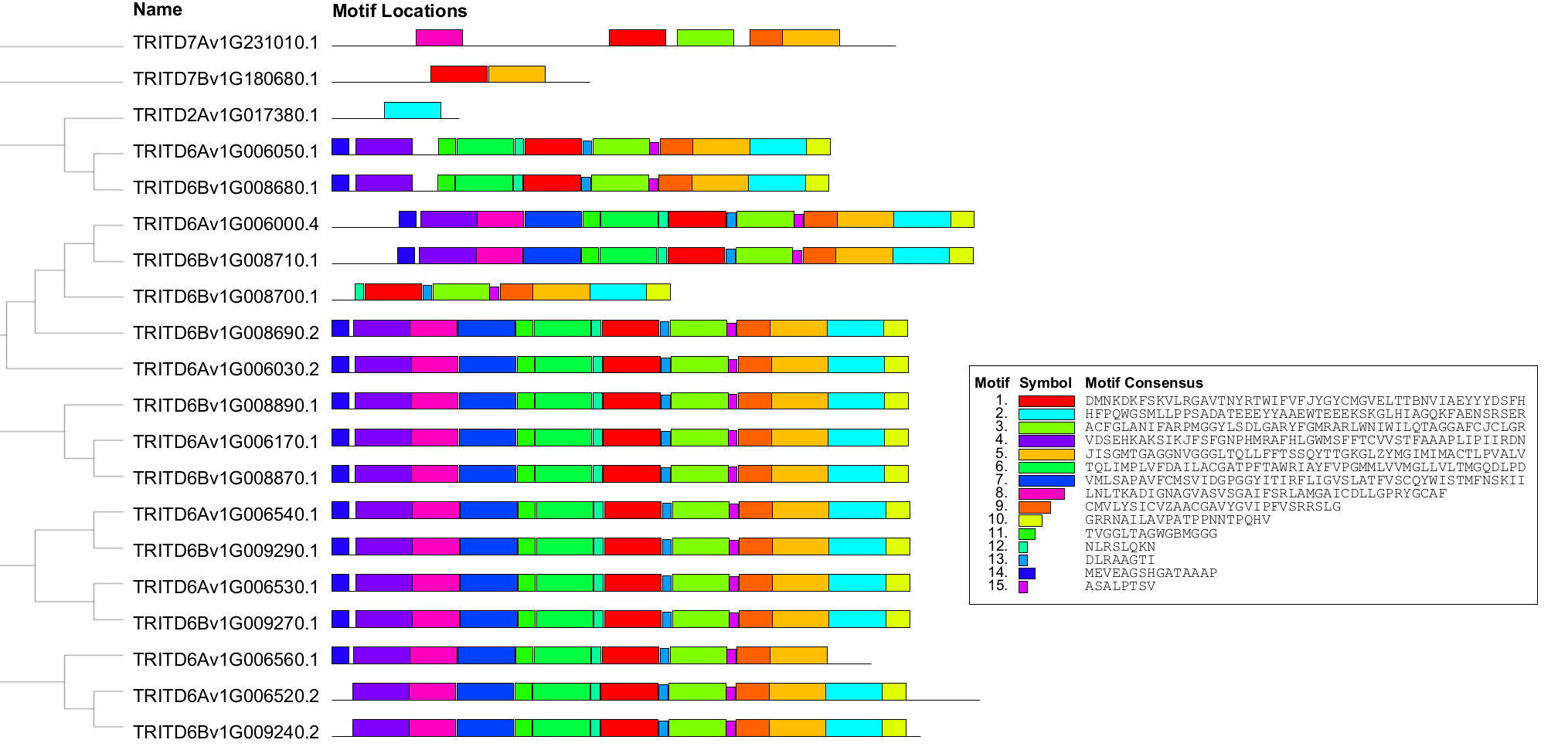
**Figure S6**. Conserved motifs detected in TdNRT2 protein sequences. The most represented motif for each of the 15 motifs is shown.


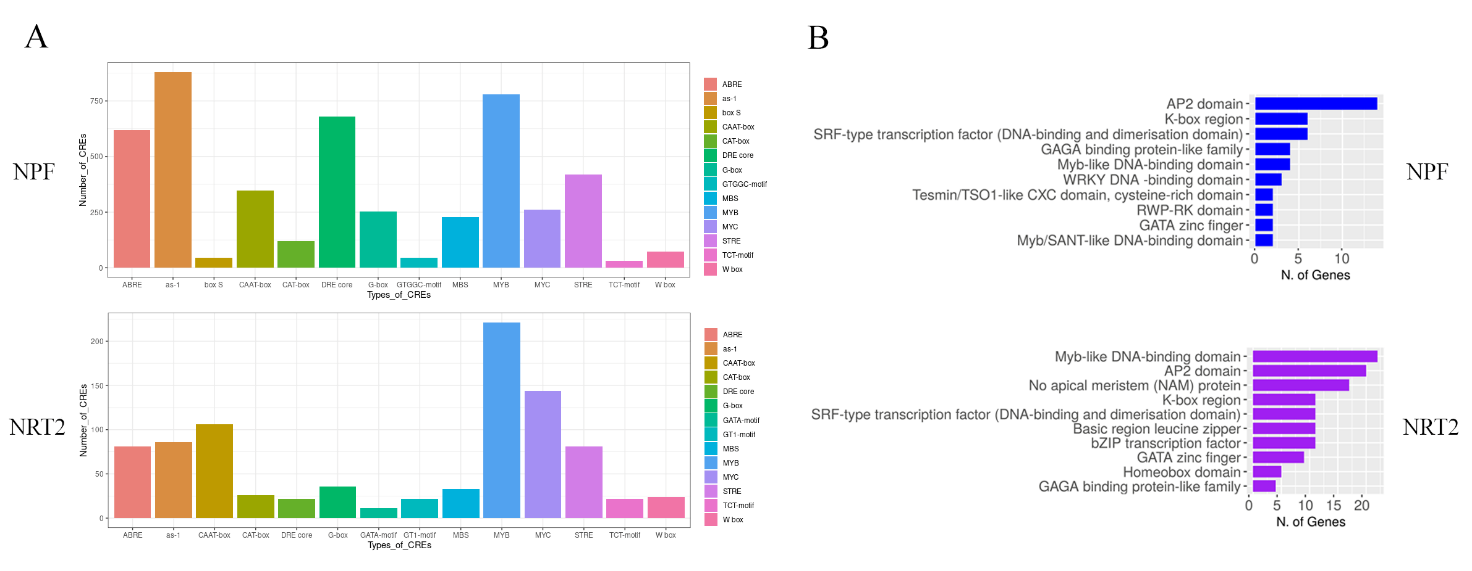


**Figure S7** Prediction of regulatory elements of both NRT2 and NPF genes. **A** Abundance of Cis-Regulatory Elements (*CRE*s) detected in the promoter regions of TdNPF and TdNRT2 genes. **B** Domains of the Transcription Factors predicted to bind to the promoter regions of TdNPF and TdNRT2 genes.


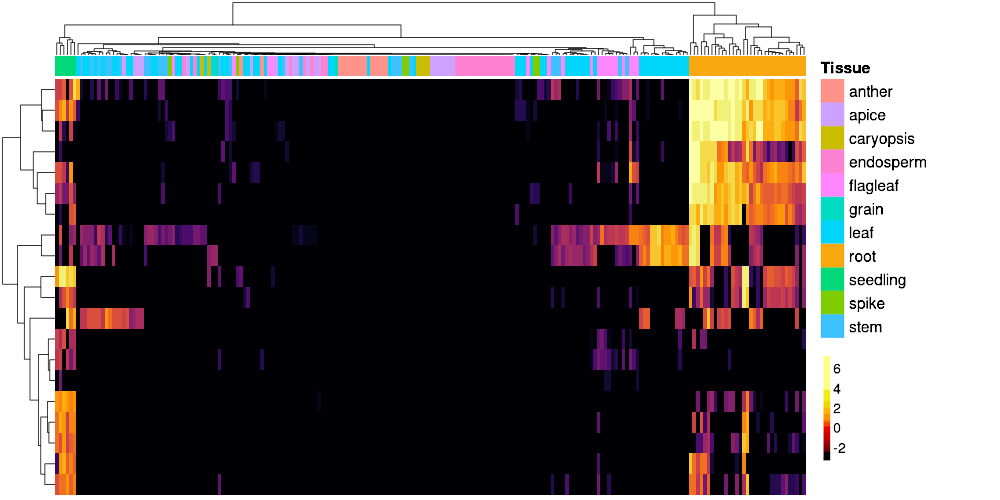
**Figure S8** Expression profiles of TdNRT2 genes in 11 tissues. Hierarchical clustering was performed both on rows and columns.


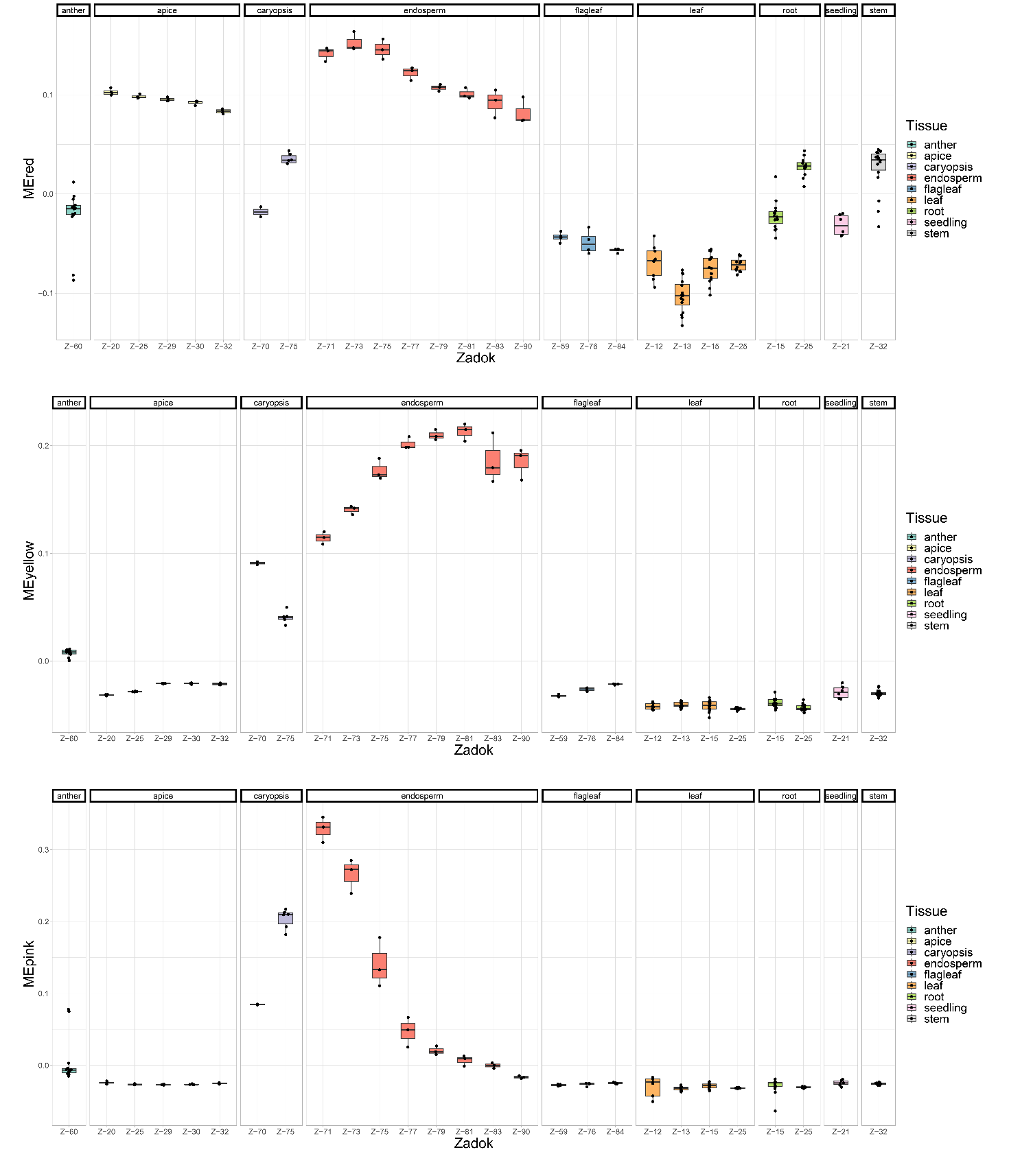


**Figure S9** Module eigengenes trend in three modules highly induced in endosperm during maturation. Red module is highly expressed during the entire maturation process, while pink module and yellow module showing almost opposite trends with the former increasing and the latter rapidly decreasing during maturation.


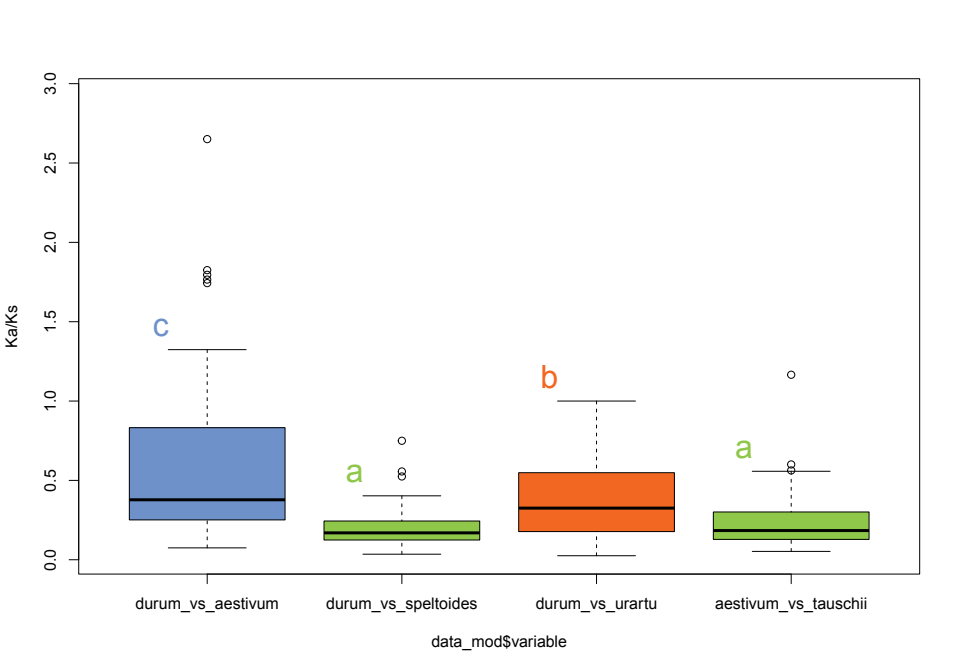


**Figure S10** Tukey test performed on the Ka/Ks for the four inter-specific comparisons
